# Supplementary material for: The Metabolism of Epoxyeicosatrienoic Acids by Soluble Epoxide Hydrolase Is Protective against the Development of Vascular Calcification
Source: Int J Mol Sci. 2020 Jun 17;21(12):4313. doi: 10.3390/ijms21124313 (PMC7352784; doi:10.3390/ijms21124313)

The metabolism of epoxyeicosatrienoic acids by soluble epoxide hydrolase is protective against the development of vascular calcification

Short title: sEH and vascular calcification

Authors: Olivier Varennes, Romuald Mentaverri, Thomas Duflot, Gilles Kauffenstein, Thibaut Objois, Gaëlle Lenglet, Carine Avondo, Christophe Morisseau, Michel Brazier, Saïd Kamel, Isabelle Six, Jeremy Bellien

SUPPLEMENTS

SUPPLEMENTARY METHODS

Pharmacological agents

Inhibition of soluble epoxide hydrolase (sEH) was obtained using *trans*-4-(4-(3-adamantan-1-yl-ureido)-cyclohexyloxy)-benzoic acid (*t*-AUCB, University of Davies, CA), added at the concentrations of 0.1, 1 and 10 µM in the culture medium. The Ki value of *t*-AUCB for the hydrolase domain of sEH is in the low nanomolar range.[1]

The role of EETs was assessed using co-treatment of aortic rings with the cytochrome P450 epoxygenases inhibitor fluconazole (100 µM; Pfizer Holding) and the EET antagonist 14,15-epoxyeicosa-5(Z)-enoic acid (14,15-EEZE: 1 µM; Sigma-Aldrich).[2,3] In addition, exogenous (±)11(12)-EET and (±)14(15)-EET (50511, 50651; Cayman Chemical Company) and the protein kinase A (PKA) inhibitor (5-24 PKI; 15996, Cayman Chemical Company) were added to the culture medium of aortic rings and/or human vascular smooth muscle cells (VSMCs).

LC-MS/MS analysis

To assess the impact of high-phosphate conditions and to monitor the impact of *t*-AUCB on sEH activity, 14,15-EET and, 14,15-DHET were quantified in the aortic culture supernatant at day 6 by liquid chromatography coupled to tandem mass spectrometry.[4] 14,15-EET and 14,15-DHET analysis was performed after protein precipitation, lipids extraction and saponification. Briefly, 300 µL of culture supernatant were spiked with 10 µL of internal standards (14,15-EETd$_8$ and 11,12-DHETd$_{11}$ at 200 ng/mL) and 1 mL of methanol was added for protein precipitation. After centrifugation, the supernatant was collected and lipids were extracted with dichloromethane after acidification with formic acid. The organic layer was evaporated to dryness under a gentle stream of nitrogen. The crude extract was saponified with sodium hydroxide at +90°C for 20 min to release fatty acids into their free form. Then, the sample was acidified with formic acid and EETs and DHETs wee extracted with a second

extraction. The sample was once again evaporated to dryness and reconstituted in 100 µL of methanol. Chromatographic separation was performed on a Kinetex C18 column (2.6-µm particle size, 50-mm length × 3-mm inner diameter). The autosampler temperature was set at 8 °C, the column oven at 30 °C, the injected volume was 20 µL, and the flow rate was 600 µL/min. The mobile phase was 0.2% formic acid in MeOH (solvent A) and 2 mM ammonium formate with 0.2% formic acid in water (solvent B). The elution was started with 95% B (0-0.5 min), 95-20% B (0.5-1 min), 20-10% B (1-4 min)10-5% B (4-5 min), 5-95% B (5-6 min), 95% B (6-7.5 min). The following multiple reaction monitoring (MRM) transitions m/z 319.1 to m/z 219.1 and m/z 337.1 to m/z 207.1 in negative ion mode were used to detect 14,15-EET and 14,15-DHET respectively.

Viability assays

Methylthiazolyldiphenyl-tetrazolium bromide (MTT; M5655, Sigma-Aldrich), a yellow tetrazole reduced to purple formazan in living cells, was used to determine the viability of aortic rings. The MTT substrate was prepared in a PBS solution at a final concentration of 0.05 mg/mL. Aortic rings were incubated in MTT solution during 1 hour at 37°C and 5% CO_2. The MTT solution was then removed and the aortic rings were left 2 hours at room temperature. DMSO was added to dissolve formazan and changes in absorbance at 570 nm, which are directly proportional to the quantity of viable cells, were measured. Trypan blue dye exclusion assays (T8154, Sigma-Aldrich) were used to determine the viability of human VSMCs. Cells were counted in 0.02% trypan blue solution and the ratio of living cells to dead cells was measured.

Quantification of calcium deposition

Aortic segments or human VSMCs were washed once with PBS and then decalcified with 0.6 N HCl overnight at 4°C. The calcium content in the HCl supernatant was colorimetrically analyzed with the o-cresolphthalein complexone method.[5] Briefly, a solution of ortho-cresolphtalein complexone at 300 μM (P5631, Sigma-Aldrich) was prepared with 8-hydroxyquinoline 16 mM (252565, Sigma-Aldrich) in 2-amino-2-methyl-1-propanol 0.5M distilled water solution (pH=10.5; Sigma-Aldrich) and heated for 2 hours. For the quantification, 25 μl of HCl supernatants were added to 150 μl of ortho-cresolphtalein solution in a 96-well plate. Plates were shacked for 15 min at 500 RPM and the absorbance was then measured at 565 nm. Calcium content in aortic rings were corrected by aortic dry weight with aortas dried overnight at 37°C.

Tissue-nonspecific alkaline phosphatase (TNAP) activity and pyrophosphate (PPi) levels

TNAP activity in the culture supernatant of aortic rings was measured, as the hydrolysis of p-nitrophenyl phosphate (pNPP), using a commercially available kit (AP0100, Sigma-Aldrich). Briefly, a pNPP solution 0.67 M was prepared in ultrapure water. In a 96 well-plate, 5 μl of cell culture supernatants were added to 240 μl of reaction buffer kit and 5 μl of pNPP solution. A blank well and a control enzyme well were used. Plates were incubated at 37°C with the absorbance at 405 nm measured each minute during 10 minutes and TNAP activity was calculated using a molar extinction coefficient of 18.75 mM/cm.

PPi concentrations in the culture supernatant of aortic rings were determined by the use of a fluorogenic pyrophosphate sensor using the manufacturer instructions (MAK168 Sigma Aldrich). Briefly, samples were diluted (1/5 assay buffer) and 20 μL were mixed with 20 μL of PPi sensor. Fluorescence was read within 15 min using microplate reader (Flexstation, Molecular Devices; λex =316/λem = 456 nm). After background signal (no PPi) removal, the concentration was determined from a PPi standard curve (10^{-7} to 10^{-4} M).

Histological assessment of vascular calcification

Samples were cryopreserved in OCT (Optimum Cutting Temperature, O.C.T Compound, Sakura® Finetek). Seven µm-thick cross sections were obtained perpendicularly to the vessels (Leica® CM 1850 Cryostat). Briefly, the slides were stained 2 minutes with Alizarin Red S solution (40 mmol/l, pH=4.2, at room temperature) to observe specifically calcium ions. Slides were then immersed successively in 3 baths (100% acetone, acetone-toluene (1:1) and 100% toluene) to dehydrate them completely. Von Kossa staining (VK) was used to identify divalent ions. The slides were fixed by ethanol with 2 successive baths for 5 minutes. A 1% silver nitrate solution was added in the dark for 30 minutes and slides were then rinsed with water. Sodium thiosulfate (5%) was added for 5 minutes, rinsed in water and Kernechtrot counterstain was added (0.2% Kernechtrot in aqueous solution, 5% aluminium sulfate) for 5 minutes. The slides were washed with water and dehydrated with 100% ethanol and toluene. Slides were mounted with DPX (44581; Sigma Aldrich) for observation with an optical microscope (Zeiss® AXIO Imager D2). Images were taken with the Histolab ® software.

Immunohistochemistry

The deendothelialization of aortic rings by gently rubbing the intimal surface of the aortic rings with a wooden stick was assessed by immunohistofluorescence using goat polyclonal IgG anti-CD31 antibody (PECAM-1; M-20 sc-1506, Santa Cruz Biotechnology). Briefly, aortic rings were fixed with 4% paraformaldehyde in PBS for 10 min at room temperature, then antigen retrieval using citrate buffer was used. Quench for 10 min with 25 mM glycine and permeabilization for 60 min at room temperature in PBS 1% BSA and 0.3 % triton X-100 were done. Aortic rings were incubated overnight at 4°C with the primary antibody (dilution 1:25 from original unit) in blocking solution. Aortic rings were washed in PBS and incubated

with secondary antibody (Alexa 488 donkey anti-goat, life technologies, A11055; dilution 1:500 from original unit) for 1 hour at room temperature. Negative control was made with secondary antibody without primary antibody. Cell nuclei were counterstained with Hoescht solution and mounted on glass using Mowiol solution for fluorescent detection.

The detection of sEH protein expression was assessed in human VSMC by immunohistochemistry using an anti-sEH antibody (sEH (A-5) sc-166961, Santa Cruz Biotechnology; dilution 1:25 from original unit).

Quantitative RT-PCR

Total RNA was extracted using the RNA isolation kit RNeasy® Mini Kit (Qiagen). Then 500 ng RNA were reversed transcribed into cDNA using the 'High Capacity cDNA Reverse Transcription Kit' (Applied Biosystems) according to the manufacturer's instructions. Primers for the osteochondrogenic transcription factor genes Runt-related transcription factor 2 (Runx2), Msh homeobox 2 (Msx2), and sex determining region Y-box 9 (Sox9), the contractile marker smooth muscle myosin heavy chain (SMMHC), sEH (EPHX2), and GAPDH were from Eurogentec (See Supplementary Table 2). The quantitative PCR was performed on Biorad CFX connect RealTime System using SYBR Green Master Mix and RNA expression levels of the different genes were corrected for GAPDH.

References

1. Tsai HJ, Hwang SH, Morisseau C, Yang J, Jones PD, Kasagami T, Kim IH, Hammock BD. Pharmacokinetic screening of soluble epoxide hydrolase inhibitors in dogs. Eur J Pharm Sci 2010;40:222-238.
2. Roche C, Besnier M, Cassel R, Harouki N, Coquerel D, Guerrot D, Nicol L, Loizon E, Morisseau C, Remy-Jouet I, Mulder P, Ouvrard-Pascaud A, Madec AM, Richard V,

Bellien J. Soluble epoxide hydrolase inhibition improves coronary endothelial function and prevents the development of cardiac alterations in obese insulin-resistant mice. Am J Physiol Heart Circ Physiol. 2015;308:H1020-H1029.

3. Gauthier KM, Deeter C, Krishna UM, Reddy YK, Bondlela M, Falck JR, Campbell WB. 14,15-epoxyeicosa-5(Z)-enoic acid: A selective epoxyeicosatrienoic acid antagonist that inhibits endothelium-dependent hyperpolarization and relaxation in coronary arteries. Circ Res. 2002;90:1028-1036.
4. Duflot T, Pereira T, Roche C, Iacob M, Cardinael, Hamza NE, Thuillez C, Compagnon P, Joannidès R,, Lamoureux F, Bellien J. A sensitive LC-MS/MS method for the quantification of regioisomers of epoxyeicosatrienoic and dihydroxyeicosatrienoic acids in human plasma during endothelial stimulation. Anal Bioanal Chem. 2017;409:1845-1855.
5. Louvet L, Büchel J, Steppan S, Passlick-Deetjen J, Massy ZA. Magnesium prevents phosphate-induced calcification in human aortic vascular smooth muscle cells. Nephrol Dial Transplant 2013;28:869-878.

SUPPLEMENTARY TABLES

Supplementary Table 1. Clinical characteristics of the 34 patients who underwent carotid endarterectomy

**Parameters**	
Age, years	70 ± 10
Gender, male/female	29/5
BMI, kg/m²	26 ± 4
SBP, mmHg	156 ± 23
DBP, mmHg	83 ± 11
Plasma glucose, mmol/L	6.0 ± 1.0
LDL, mmol/L	2.7 ± 1.0
HDL, mmol/L	1.0 ± 0.3
Triglycerides	1.8 ± 1
Hypertension, %	76
Diabetes, %	38
Hypercholesterolemia, %	79

Results are means ± SD for continuous parameters

Supplementary Table 2. Primers used for quantitative RT-PCR

	**Forward (5'-3')**	**Reverse (3'-5')**
GAPDH	TATGATGACATCAAGAAGGTGG	CACCACCCTGTTGCTGTA
Runx2	CCTCTGACTTCTGCCTCTGG	GATGAAATGCCTGGGAACTG
Msx2	CAGAACCGAAGGGCTAAGGCA	GTAGGAGGCGCTGTATATGG
Sox9	GTGCTGAAGGGCTACGACTGG	GCAGATGCGGGTACTGGT
SMMHC	CAGTATTTGGCTGTGGTGGC	CATTGCCGAAAGCCTCCAGG
EPHX2	AAGATTTAGCCAGTGGCGTGTC	ATCACTGCTGGCAAAAGAACG

SUPPLEMENTARY FIGURES

Supplementary Figure 1. Relative calcium content of aortic rings cultured during 7 days in 0.9 mM inorganic phosphate (Pi) in absence and in presence of 10 µM t-AUCB (B; n=3 per group).

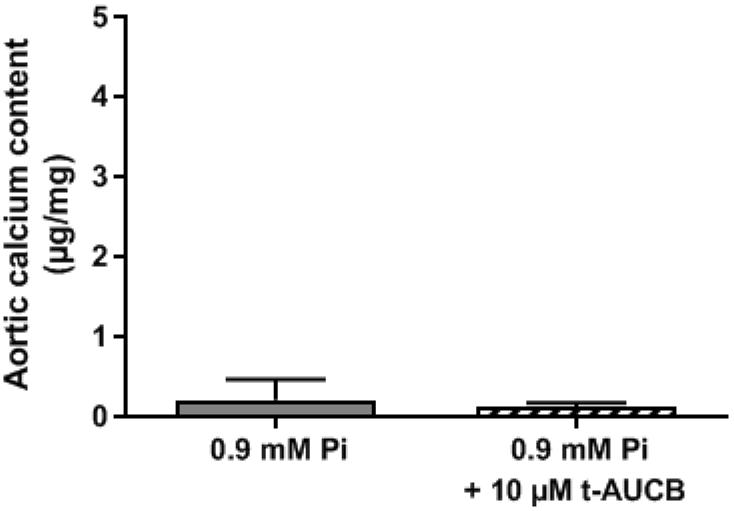

Supplementary Figure 2. Impact of 10 µM *t*-AUCB alone and combined with 100 µM fluconazole (n=5-11 per group) on the viability of rat aortic rings assessed using a methylthiazolyldiphenyl-tetrazolium assay.

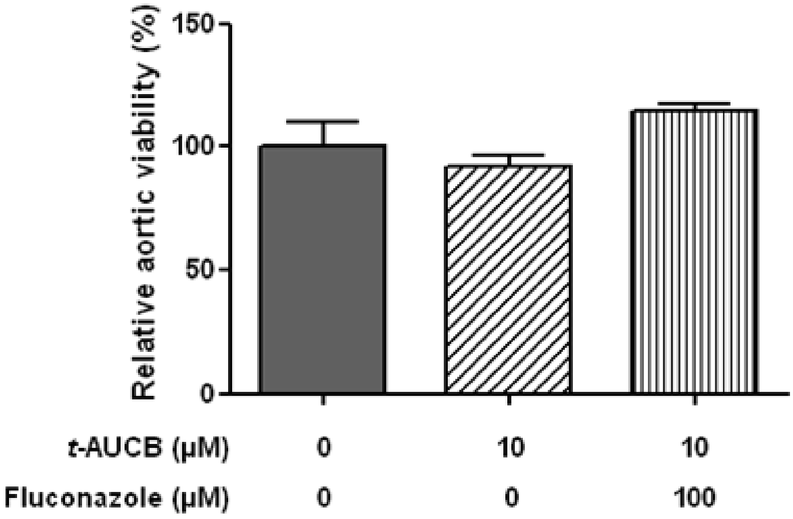

Supplement: Supplementary file 1 [file ijms-21-04313-s001.pdf]
